# Seroprevalence and Risk Factors of African Horse Sickness in Three Agroecological Zones of Cameroon

**DOI:** 10.1155/2022/2457772

**Published:** 2022-05-14

**Authors:** Mohamed M. F. Ndebé, Mohamed M. M. Mouiche, Frédéric Moffo, Rodrigue N. S. Poueme, Julius Awah-Ndukum

**Affiliations:** ^1^Institute of Agricultural Research for Development, Bangangté Polyvalent Station, P.O. Box 222, Bangangté, Cameroon; ^2^Department of Animal Science, Faculty of Agronomy and Agricultural Sciences, University of Dschang, B.P. 222, Dschang, Cameroon; ^3^School of Veterinary Medicine and Sciences, University of Ngaoundéré, P.O. Box 454, Ngaoundéré, Cameroon; ^4^National Veterinary Laboratory, Garoua, Cameroon; ^5^Department of Animal Production Technology, College of Technology, University of Bamenda, P.O. Box 39, Bambili, Cameroon

## Abstract

African horse sickness (AHS), a highly fatal arbovirosis of equines is endemic in sub-Saharan Africa. However, its epidemiology is poorly known in Cameroon. This study aimed to investigate the prevalence profile and risk factors of African horse sickness in Cameroon. Horse sera were subjected to the ELISA blocking test for the determination of antibodies against African horse sickness virus, and positive samples were submitted to capture ELISA to determine the presence of antigens. Potential risk factors associated with AHS were assessed based on the information collected in the field. The chi-square test and the odd ratio (OR) were used to test the association between serology and the different variables. Of the 336 sera obtained, 198 were positive for antibodies with a prevalence of 58.93% (CI: 53.67–64.19). From the 198 positive sera to antibodies, only one revealed positivity to antigens with a prevalence of 0.51% (CI: 0–1.5). Agroecological zone I (94.31%, CI: 91.83–96.79, OR: 34.92) was significantly (*p* < 0.05) associated with the higher risk of disease dissemination than agroecological zone II (66.67%, CI: 61.63–71.71, OR: 4.21) and agroecological zone III (32.18%, CI: 27.18–37.18; OR: 1). Males (63.59%, CI: 58.44–68.74, OR: 1) were significantly (*p* < 0.05) affected than females (50.42%, CI: 45.07–55.77; OR: 0.58). Horses of more than 8 years (76.00%, CI: 71.43–80.57) were significantly (*p* < 0.05) at risk than young animals of less than 3 years old (32.14%, CI: 27.15–37.13, OR: 0.15). This study highlights a high seroprevalence of antibodies of African horse sickness in Cameroon. Agroecological zone, age, and the importation of horses were highly associated with the distribution of disease at the national level.

## 1. Introduction

African horse sickness (AHS) is a highly fatal arbovirosis of equines caused by a virus of the genus *Orbivirus*, family Reoviridae, and possess 9 serotypes [1] with 84 strains [2]. AHS affects all equine species [3, 4], solipeds [4], and accidentally carnivores [5–8]. The main route of transmission is indirectly via *Culicoides*. However, it can also be transmitted iatrogenically via blood products or tissue suspension [9–12] with the aid of mosquitoes and mechanical vectors of the genuses *Stomox* and *Tabanidae* [6].

AHS is a noncontagious disease which affects the respiratory and circulatory functions [3] with an incubation period of approximately 3 days to 2 weeks [13]. AHS is manifested either in a pulmonary form (characterized by fever, respiratory distress, foamy discharge, sweating [1], animal in a motionless state with lingering tongue, fore-limbed, extended on the neck, arched back with a death rate of 95% within 24–48 hours [9]) or in cardiac form (characterized by fever [1], swelling of the face, tongue, intermandibular space, laryngeal region, and sometimes the neck, shoulder, and chest [13] and may even reach the lungs [10]). Esophageal paralysis, optic nerve palsy [14], and sublingual haemorrhage are at an unfavourable prognosis with 50% of mortality [3].

Although the lesions and clinical signs are characteristic, laboratory diagnosis of AHS is essential as it can be confused with equine influenza, bacterial pneumonia, anthrax, equine congestive failure, granulocytic ehrlichiosis equine [15], viral arteritis, equine infectious anemia, hemorrhagic purpura [1], and equine encephalosis [16]. So far, there is no treatment for African horse sickness [1], but nevertheless, preventive measures can be taken to control the incidence of the disease via health prevention in an infected environment or in a healthy environment [10, 16, 17] and/or medical use of vaccines [1, 18]. The presence of *Culicoides*, mosquitoes, and ticks [19] and their periods of activity during the warm and wet season [20] increase the incident of AHS. However, mild and humid climates, wet and muddy soils, and high temperatures favour the presence of these insects [19]; thus, the disease is found mainly in low and humid areas near water points, where the vegetation cover maintains hygrometric conditions favourable to larval development and adult survival. The climatic conditions in sub-Saharan Africa facilitates the permanent survival of vectors throughout the year [21] and the use of live vaccines in nonenzootic areas (risk of reversion to virulent strains) [16]. AHS is endemic in sub-Saharan Africa, and the estimated prevalence of antibodies in horses of 86.6% [22], 81.% [23], and 46.% [24] was reported in Nigeria, Gambia, and Ethiopia, respectively. In Cameroon, there is little or no information regarding the profile of AHS. Therefore, this study was carried out as part of the national surveillance program to provide knowledge on the epidemiology of AHS and the factors contributing to the spread of disease in Cameroon.

## 2. Materials and Methods

### 2.1. Study Area and Study Design

The present cross-sectional study was carried out from May 2016 to April 2017 in 3 agroecological zone of Cameroon including Sudano-Sahelian zone or zone I which regrouped two regions, namely, far north (10°–12° N and 14°–15° E) and north (8°–10° N and 12°–14° E) regions, Guinean savannah zone or zone II (Adamawa: 5°–8° N and 11°–14° E), and Western highlands zone or zone III with west (17'54” N and 10°22'32”E) and northwest (6°22'0”.01 N and 10°22'0”.01 E) regions. These 3 agroecological zones contribute over 98.35% of the national horse herd. Sudano-Sahelian zone had low altitudes, highest peak of which is 1442 m, an average temperature of 28°C (maxima of 40–45°C), and a monomodal rainfall of 400–1200 mm per year. The high Guinean savannah zone is characterized by a vast plateau of altitudes between 900 and 1500 m and the climate is of the Sudanese, humid tropical type with two seasons per year. The average annual rainfall is about 1500 mm; the temperatures are moderate, with an average of 20–26°C. The Western highlands zone is characterized by a low mean temperature (19°C) and heavy rains fall of 1500–2000 mm per year [25].

### 2.2. Sample Size and Data Collection

A minimum sample size of 179 horses was estimated [26] based on previous reports on the prevalence of AHS of 86.6% in Nigeria [22] with a confidence interval of 95% and precision set at 5%. The study used a stratified random technique. Sampling of each region was according to their proportion in the national herd size. The random number generation technique was used for the selection of herd from a list obtained at the Delegations of Livestock, Fisheries, and Animal Industries (DREPIA) in the study regions and completed by private field veterinary practitioners. The Scientific Research and Ethics Committee of the School of Veterinary Medicine and Sciences of the University of Ngaoundere, Cameroon (2017/017/UN/ESMV/D), provided ethical approval for this research. Following explanation of the purpose of the study to horse owner in these regions, they provided written consents before they and their animals were included in the study. The information related to the horse were collected.

Apart from procedural restraining manipulations for safety purposes and jugular venipuncture for blood sampling (>15 mL) using sterile vacutainer, the animals were not subjected to suffering. Briefly, for each selected animal, blood samples were collected in a test tube containing ethylene diamine tetra-acetic acid and transported to the National Veterinary Laboratory, Bokle Garoua, Cameroon. Blood samples were centrifuged, and sera obtained were used for detection of the antibodies using ELISA blocking (INgezim AHSV Compac Plus Kit; Ingenasa, Madrid, Spain). All the positive sera were further subjected to capture ELISA using the INgezim AHS DAS Kit (Ingenasa, Madrid, Spain) for antigen detection.

### 2.3. Data Analysis

Data entry was performed with Microsoft Excel 2013 (Microsoft Corporation, Redmond, WA, USA). Descriptive statistics comprising percentages were used to indicate the prevalence of AHS in these five regions. The chi-square test was used to test significant levels within factors on seroprevalence rates, and odd ratios (OR) were determined for associated risk factors along 95% confidence intervals and statistical significance set at *p* < 0.05. All data were computed using IMB SPSS Statistics (ver. 21.0).

## 3. Results

### 3.1. Distribution of Samples Collected

Of 336 samples collected, more than half (51.8%) were from the Western highlands agroecological zone, and northwest region (39.0%) had the higher number of samples. Horse aged between 3 and 8 years old (75.9%) were mostly investigated, with male (64.6%) being the commonly encountered animals. Horse were mostly imported from Nigeria (98.21%) (Table 1).

### 3.2. Seroprevalence of African Horse Sickness in the Study Area

Of the 336 sera obtained, 198 were positive for antibodies with a prevalence of 58.93% (95% CI: 53.67–64.19). From the 198 positive sera to antibodies, only one revealed positivity to antigens with a prevalence of 0.51% (95% CI: 0–1.5). Prevalence was significantly different (*p* < 0.001, *χ*^2^ = 18.49) from one region to another. Seroprevalence were higher in the north (96.05% (95% CI: 93.97–98.13) and far north regions and 91.49% (95% CI: 88.51–94.47), respectively) than the northwest region (25.95% (95% CI: 21.26–30.64)) (Table 2).

### 3.3. Factors Affecting the Distribution of African Horse Sickness in the Study Area

Agroecological zone I (94.31% (95% CI: 91.83–96.79, OR: 34.92)) was significantly (*p* < 0.05) associated with the higher risk of disease dissemination than agroecological zone II (66.67% (95% CI: 61.63–71.71, OR: 4.21)) and agroecological zone III (32.18% (95% CI: 27.18–37.18; OR: 1)). Males (63.59% (95% CI: 58.44–68.74, OR: 1)) were significantly (*p* < 0.05) affected than females (50.42% (95% CI: 45.07–55.77; OR: 0.58)). Horses of more than 8 years (76.00% (95% CI: 71.43–80.57)) were significantly (*p* < 0.05) at risk than young animals of less than 3 years old (32.14% (95% CI: 27.15–37.13, OR: 0.15)) (Table 3).

## 4. Discussion

This study was carried out with aim to evaluate the epidemiological situation of African horse sickness in Cameroon. The investigation indicate that the disease is highly prevalent in the study area. AHS virus-specific antibody was more detected than the specific antigen and may be justified by a recent infection of less than 90 days of the animals [27]. Horses recovering from natural infection develop strong immunity against the same serotypes [4] and may develop partial immunity against heterologous serotypes [6] within 8–12 days postinfection, while vaccination can protect for up to 4 years [28] and explains why more horses positive for antibodies were antigen negative. In Africa, antigen detection has only been reported during epizootics or suspected cases of AHS. Contrary wise, many studies have reported the prevalence of antibodies with results comparable to that obtained in this work. Indeed, seroprevalence was higher than that obtained in Ethiopia (46%) [24], but lower than 86.6% reported in Nigeria [22], 91.81% in Senegal [12], and 81% in Gambia [23]. The difference may be related to the variation in agroecological contexts and the control strategy develops in each country.

Considering the scale of agroecological zones of Cameroon, the seroprevalence was higher in the agroecological zone I (low altitude zone), agroecological zone II (average altitude), and the agroecological zone III (high altitudes). This is an indication that altitude influences the distribution of the disease. Disease distribution was more prevalent in low altitudes than in high altitudes, and this corroborates with previous findings reported in Ethiopia [24]. In this study, prevalence was higher in hot and dry savannah areas, which is consistent with that reported in Nigeria [22], and indicates that climatic conditions favoured with the propagation and transmission of the virus. Since AHS is mainly transmitted by *Culicoides*, an agroecological zone constitutes potential risk factors as it favours the survival and multiplication of insects. Thus, the lower the temperature, the longer the durations of the different life stages, thus making viral replication impossible or totally inactive in the insects [29]. High temperatures increase the lifespan of adults [30]. In addition, hotter periods coupled with heavy rains conditions increase the reproduction and activity of *Culicoides* [31]. Similarly, a predominance of *Culicoides* in semihumid areas has been observed and their numbers drastically decreased in respect of rainfall intensity [32]. Nevertheless, a decrease in temperature and increase in rainfall from zone I to zone III explains the decrease in prevalence in the same direction. In addition, sandy zones constitute a suitable habitat for the burial of *Culicoides* [32], which is the case of the agroecological zone I. In the agroecological zone I, many horses were imported from Nigeria where several outbreaks of AHS were reported [22]. In addition, the regular participation of horses from the north and far north in equine competition in this neighboring country could contribute to the increase of the prevalence in these regions. Horses raising in the modern system were at greater risk than those in the traditional system and males were more at risk than in females. This result was different to that reported in Ethiopia [24, 33]. Males are preferentially used by shepherds to accompany cattle in search of pastures or during transhumance and results with the increasing risk of contact with the wild virus. On the other hand, males are more involved in many competitions and organized activities in Chad and Nigeria. Almost, 98.21% of the horses imported in Cameroon are males from Nigeria and Chad, where the disease is highly prevalent [10]. Young animals of less than 3 years old were less at risk compared to older horses. Despite the possible loss of maternal immunity, some foals do not come in contact with the virus, while older horses have more chance to get in contact to the virus. Similar observations were reported by Bazarusanga in Senegal [5] where old animals were more likely to come into contact with the virus.

The absence of a vaccination program against AHS in Cameroon coupled with the fact that it is a vectorborne disease and testifies not only to natural infection but also to the presence and activity of vectors. The absence of a vaccination program coupled with the fact that no case of AHS has ever been reported in Cameroon may be in line with the circulation of wild strains with naturally attenuated virulence or not very virulent. Howell established in 1962 that strains belonging to serotype 9 is the least virulent and only serotype 9 was historically identified in Central Africa [34], including Cameroon. However, these strains could also be attenuated by evolution as a result of repeated infections of a naive population that has been in contact with the wild strain for several years, which is in accordance with the hypothesis of Oura et al. [35]. This could also be as a result of the use of old strain in vaccine in neighboring countries. Another hypothesis would be a state of endemic stability to the serotypes circulating following the infection of the foals at an early age (production of natural antibodies), whereas the latter still possesses partially or totally antibodies inherited from maternal colostrum [35]. The last and most important hypothesis would be the existence of cross reactions with one or more unidentified orbiviruses.

The low antigen detected in this study would be due to the concentration of VP7 protein that did not reach the detectable threshold (15 ng/ml) in the serum with the ELISA kit used. In addition, the detection rate of viral antigen using ELISA showed a higher rate in blood [18]. Since the virus is firmly bound to the surface or to the interior of red blood cells [36, 37], the use of hemolyzed blood instead of serum will give better results. However, a study conducted in Nigeria [37] states that the detection rate of circulating lymphocyte antigens by the ELISA method is higher than that of hemolyzed red blood cells. For this purpose, the detection of antigen by ELISA from circulating lymphocytes would be ideal for future studies. Real-time PCR, which is a highly sensitive and specific method, could be a method of choice for future studies on the subject because it can detect very little amount of viral RNA [3] and can give better results as the viral genome can be detected up to 120 days after infection [38].

## 5. Conclusion

This study highlights that African horse sickness virus circulated in Cameroon with a high prevalence. Agroecological zone and sex are being the most influencing factors. Continuous surveillance is important for decision making to reduce the burden of African horse sickness in animal health in Cameroon.

## Figures and Tables

**Table 1:** Distribution of samples collected in the study area.

Factors	Variables	Number of samples (%)
Agroecological zone	Sudano-Sahelian (zone I) Guinean savannah (zone II) Western highlands (zone III)	123 (36.6) 39 (11.6) 174 (51.8)

Regions	Adamawa Far north North Northwest West	39 (11.6) 47 (14.0) 76 (22.6) 131 (39.0) 43 (12.8)

Age (year)	<3 (3–8) ≥8	56 (16.7) 255 (75.9) 25 (7.4)

Sex	Male Female	217 (64.6) 119 (35.4)

Sources of imported horses	Nigeria Chad	110 (98.21) 2 (1.77)

**Table 2:** Seropositivity of African horse sickness among the horses in the study area.

Category	Variable	Number (positive)	Seropositivity (%) (95% CI)	*χ* ^2^ (*p* value)
Regions	Adamawa Far north North Northwest West	39 (26) 47 (43) 76 (73) 131 (34) 43 (22)	66.67 (61.63–71.71) 91.49 (88.51–94.47) 96.05 (93.97–98.13) 25.95 (21.26–30.64) 51.16 (45.82–56.50)	18.496 (<0.001)

Age (year)	<3 (3–8) ≥8	56 (18) 255 (161) 25 (19)	32.14 (27.15–37.13) 63.14 (57.98–68.30) 76.00 (71.43–80.57)	21.22 (0.21)

Sex	Female Male	119 (60) 217 (138)	50.42 (45.07–55.77) 63.59 (58.44–68.74)	5.511 (0.02)

Husbandry system	Modern Traditional	24 (21) 37 (21)	87.50 (83.96–91.04) 56.76 (51.46–62.06)	6.416 (0.02)

**Table 3:** Factors affecting the dissemination of African horse sickness in Cameroon.

Category	Variable	Number (positive)	Seropositivity (%) (95% CI)	*χ* ^2^ (*p* value)	Odd ratio (95% CI)
Age (year)	<3 (3–8) ≥8	56 (18) 255 (161) 25 (19)	32.14 (27.15–37.13) 63.14 (57.98–68.30) 76.0 (71.43–80.57)	21.22 (0.001) 21.22 (0.21)	0.15 (0.05–0.44) 0.5 (0.21–1.40)

Sex	Female Male	119 (60) 217 (138)	50.42 (45.07–55.77) 63.59 (58.44–68.74)	5.511 (0.02)	0.58 (0.37–0.92)

Agroecological zone	Zone I Zone II Zone III	123 (116) 39 (26) 174 (56)	94.31 (91.83–96.79) 66.67 (61.63–71.71) 32.18 (27.18–37.18)	116 (0.001) 116 (0.001)	34.92 (15.28–79.80) 4.21 (2.012–8.81) 1

Husbandry system	Modern Traditional	24 (21) 37 (21)	87.50 (83.96–91.04) 56.76 (51.46–62.06)	6.416 (0.02)	5.33 (1.35–21.06) 1

## Data Availability

The datasets used during the current study are available from the corresponding author upon request.
